# Machine learning-based prediction of surface quality and tool performance in the grinding of inconel 800

**DOI:** 10.1038/s41598-025-28103-5

**Published:** 2025-12-01

**Authors:** Ramdev P. Menon, K. Abhishek, M. Vishnu, T. Satish Kumar, A. Sumesh, Ranjan Kumar Ghadai, Kanak Kalita

**Affiliations:** 1 Department of Mechanical Engineering, Amrita School of Engineering, Amrita Vishwa Vidyapeetham, Coimbatore, 641112 India; 2 Department of Mechanical and Industrial Engineering, Manipal Institute of Technology, Manipal Academy of Higher Education, Manipal, 576104 India; 3 Department of Mechanical Engineering, Rajalakshmi Institute of Technology, Chennai, 600124 India

**Keywords:** Inconel 800, Precision grinding, Tool wear prediction, Regression modeling, Infrared thermal monitoring, Engineering, Materials science

## Abstract

This study investigates the surface grinding behavior of Inconel 800, a nickel-based superalloy widely used in high-temperature applications. Grinding tests were performed using green silicon carbide and aluminium oxide wheels under constant parameters: 2800 RPM spindle speed, 0.1 mm depth of cut, and 3.5 mm feed rate, with bio-based coolant. Surface roughness was monitored after every two passes, along with corresponding thermal imaging and wheel surface analysis. Results showed that the green silicon carbide wheel maintained better thermal stability and wear resistance, with surface roughness rising from <0.3 µm to >0.85 µm by the 22^nd^ pass. In contrast, the aluminium oxide wheel delivered a finer initial finish but wore more rapidly due to heat buildup. Manually annotated particle accumulation data enabled the development of machine learning models for tool wear prediction, with Random Forest Regression achieving the highest accuracy (R^2^ >0.9). The findings highlight the effectiveness of combining thermal and surface data with predictive modeling to optimize grinding performance and tool life in machining Inconel 800.

## Introduction

The ability of nickel-based superalloys to maintain structural stability, mechanical strength, and resistance to corrosion under extreme mechanical and thermal loads makes them popular in advanced engineering applications^1–4^. These characteristics make them appropriate for parts used in vital industries like nuclear energy, petrochemicals, and aerospace, where materials must function dependably in the face of high temperatures, corrosive environments, and changing stress levels. Of these alloys, Inconel 800 is particularly prized for its durability under extended heat exposure, resistance to oxidation, and stability at high temperatures^5,6^. It is a good choice for parts that need to last for a long time in demanding service environments because of these qualities. But when it comes to machining, Inconel 800 presents considerable difficulties. Cutting forces and energy requirements during material removal are increased by its high strength at high temperatures. Its low thermal conductivity also prevents heat from dissipating at the tool-workpiece interface, which causes localized overheating and rapid tool wear. Additionally, the alloy has a tendency to harden under deformation, which increases cutting resistance and speeds up tool deterioration. Achieving high surface quality and fine dimensional accuracy is challenging due to these factors combined. For parts used in the aerospace and medical industries, where performance and dependability are crucial, maintaining surface integrity is especially important, especially with regard to residual stress, hardness, and subsurface microstructure^7^.

A successful method for cutting intricate profiles in heat-resistant alloys is high-efficiency deep grinding, or HEDG. It has been effectively used, for instance, to create fir tree root forms in turbine blades composed of superalloys such as DZ125. According to experimental findings, increasing the grinding wheel speed preserves the subsurface microstructure while improving efficiency, lowering specific energy consumption, and improving surface finish. HEDG can create surfaces with roughness values as low as 0.6 µm when it is operated at speeds greater than 80 m/s. Localized heat damage is less likely at these speeds because thermal distribution is consistent throughout the workpiece^8^. Several studies have investigated alternative cooling and lubrication strategies to enhance the grinding performance of Inconel 718. One promising method involves the use of micro-quantity soap-water mixtures, which has shown better process stability, lower grinding forces, and improved surface finish compared to both dry and conventional flood cooling. Notably, these results were achieved with minimal power consumption, reportedly as low as 10 watts^9^. Numerical modelling has been used in conjunction with experimental advancements to gain a better understanding of Inconel 718’s mechanical and thermal behavior during grinding. The Johnson–Cook constitutive model has been used in Finite Element Method (FEM) simulations to examine material removal behavior, temperature distribution, and stress concentration. When optimized conditions are used, these simulations have demonstrated a 25% reduction in surface roughness and residual stress, which has been confirmed by experiments and statistical tools such as the Taguchi method^10^.

Both surface quality and tool life are significantly influenced by the grinding wheel itself. The performance of various abrasives was compared in studies involving Udimet 720, another nickel-based superalloy. While diamond wheels produced smoother surfaces with average Ra values of 0.75 µm, cubic boron nitride (CBN) wheels showed superior resistance to wear. Furthermore, reducing wheel degradation without sacrificing the workpiece’s thermal or microstructural integrity was made possible by raising the spindle speed from 60,000 to 90,000 rpm^11^. In turning operations involving Inconel 718, edge wear on CBN cutting inserts was predominant, even under flood-cooled conditions. Here, tool degradation was primarily influenced by cutting speed and insert material, highlighting the sensitivity of tool wear to process parameters^12^.

Advancements in grinding wheel conditioning and preparation have also enhanced process precision and surface quality. The integration of ultra-hard abrasives, innovative bonding materials, and non-traditional conditioning methods—such as Electrolytic In-Process Dressing (ELID)—has enabled the achievement of ultra-smooth surfaces, in some cases exhibiting roughness values below 1 nm^13^. A detailed parametric investigation into the grinding of Inconel 718 demonstrated that grinding wheel speed and abrasive grit size played dominant roles in determining surface quality. Notably, the traverse speed of the workpiece exhibited minimal influence. Optimal grinding conditions were found using a green silicon carbide wheel (#80 mesh) operating at a speed of 30 m/s. Under these parameters, the resulting surface finish showed minimal damage and consistent quality, highlighting the abrasive’s suitability for precision operations on nickel-based alloys^14^.

In monitoring tool wear, recent developments in sensing have made real-time evaluation more reliable. For example, grayscale image features captured from CBN wheels using high-resolution imaging were analyzed with support vector regression (SVR) models. The predictions closely matched actual wear levels, with a reported correlation coefficient (R^2^) of 0.959, confirming the method’s accuracy^15^.Efforts to improve grinding process monitoring have combined visual inspection with digital techniques. One study applied image processing tools in MATLAB to examine chip adhesion on vitrified CBN wheels. Techniques such as edge detection, morphological filtering, and image normalization were used to quantify wheel loading. These observations were validated through scanning electron microscopy (SEM), showing a clear relationship between chip buildup, cutting conditions, and material removal^16^. Apart from visual inspection, acoustic emissions have also been used to assess wheel condition. In one approach, grinding noise signals were analyzed using wavelet transformations. This enabled automated classification of wheel wear stages, reaching up to 97% accuracy—without manual supervision. Such non-contact monitoring techniques offer practical advantages in automated production environments^17^. Advances in grinding wheel design have also contributed to improved performance. Wheels with multi-grit structures were tested on Inconel 625 and showed reduced surface chipping and better surface uniformity. This improvement was linked to optimized spacing between abrasive grains and more effective chip evacuation^18^.

Sinha et al investigated the grindability of Inconel 718^19^ and Inconel 625^20^, both of which are difficult-to-grind superalloys, using conventional abrasive grinding wheels and minimum quantity lubrication (MQL) grinding. For Inconel 718, the study compared silicon carbide (SiC) and alumina (Al₂O₃) wheels under dry conditions, utilizing surface characterization techniques such as EDX, XRD and XPS. The results showed that alumina wheels provided better grinding efficiency, whereas SiC wheels suffered from severe attritious wear due to chemical reactions at higher grinding temperatures. In the case of Inconel 625, the study used Box-Behnken design to optimize MQL grinding parameters like tangential force, surface roughness, specific energy, and coefficient of friction. Machine learning techniques, specifically Random Forest Regression and Gaussian Process Regression (GPR), were employed, with GPR proving superior in predictive accuracy. Multi-criteria decision-making methods, including TOPSIS and VIKOR, identified the optimal grinding conditions, resulting in low tangential force, low specific energy, and high surface roughness. SEM and EDS analysis of the ground surfaces confirmed the effectiveness of the optimized MQL grinding conditions, highlighting the benefits of MQL in maintaining grit sharpness over longer periods.

Kamal Kishore et al.^21^ investigated the influence of lubrication strategies on the grinding of Inconel 625 under dry, wet, and MQL environments. Their study combined response surface methodology and multiple ML models to predict tangential forces and surface roughness. The KNN model exhibited superior accuracy (R^2^ >0.95), while MQL grinding demonstrated reduced forces and acceptable surface finish, confirming its potential as a sustainable substitute for conventional methods.

In predictive modeling, soft computing techniques such as artificial neural networks (ANNs) and adaptive neuro-fuzzy inference systems (ANFIS) have been applied to forecast surface roughness and material removal rates during grinding of Inconel 800. Among these, ANFIS delivered the most accurate predictions, suggesting its effectiveness in modeling nonlinear process behavior^22^. Real-time wear tracking has also been achieved using line-scan cameras and linear CCD systems. Features such as kurtosis and entropy were extracted from captured images and processed using neural networks to estimate the number of grinding passes completed. These systems demonstrated prediction errors below 5%, making them well-suited for high-volume production settings^23^.

Recent imaging and sensing methods have further improved the ability to detect wheel clogging, thermal degradation, and wear. These tools reduce dependence on empirical tuning and enable a more controlled grinding process^24^. Analytical models have been proposed to explain wheel loading caused by metal adhesion. These models take into account cutting speed, depth of cut, and the structure of the abrasive. Experimental results show that most clogging occurs shortly after wheel dressing and stabilizes with continued use, reinforcing the need for careful control of process parameters^25^.

In studies focused on surface grinding of Inconel 800, grinding variables and abrasive selection were found to significantly affect surface morphology and residual stresses. Using 3D profilometry, XRD, SEM, and optical microscopy, vitrified silicon carbide wheels were shown to outperform Al₂O₃ wheels, especially under wet conditions. The lower surface roughness observed was attributed to better control of elastic–plastic deformation mechanisms^26^. Building on these foundations, the present study proposes a unique framework integrating manual surface annotation, infrared thermal profiling, and machine learning. Inconel particles adhered to the grinding wheel are marked manually across six defined regions at regular intervals—specifically, after every two grinding passes. Simultaneously, temperature data are captured using thermal imaging. The collected data are used to train regression-based models capable of predicting tool wear, optimal dressing intervals, and grinding wheel lifespan. This study aims to establish a cost-effective, data-driven decision system for process optimization, relying solely on surface quality and thermal response—without requiring invasive sensing or post-process inspection.

## Materials and methods

**Fig. 1:**
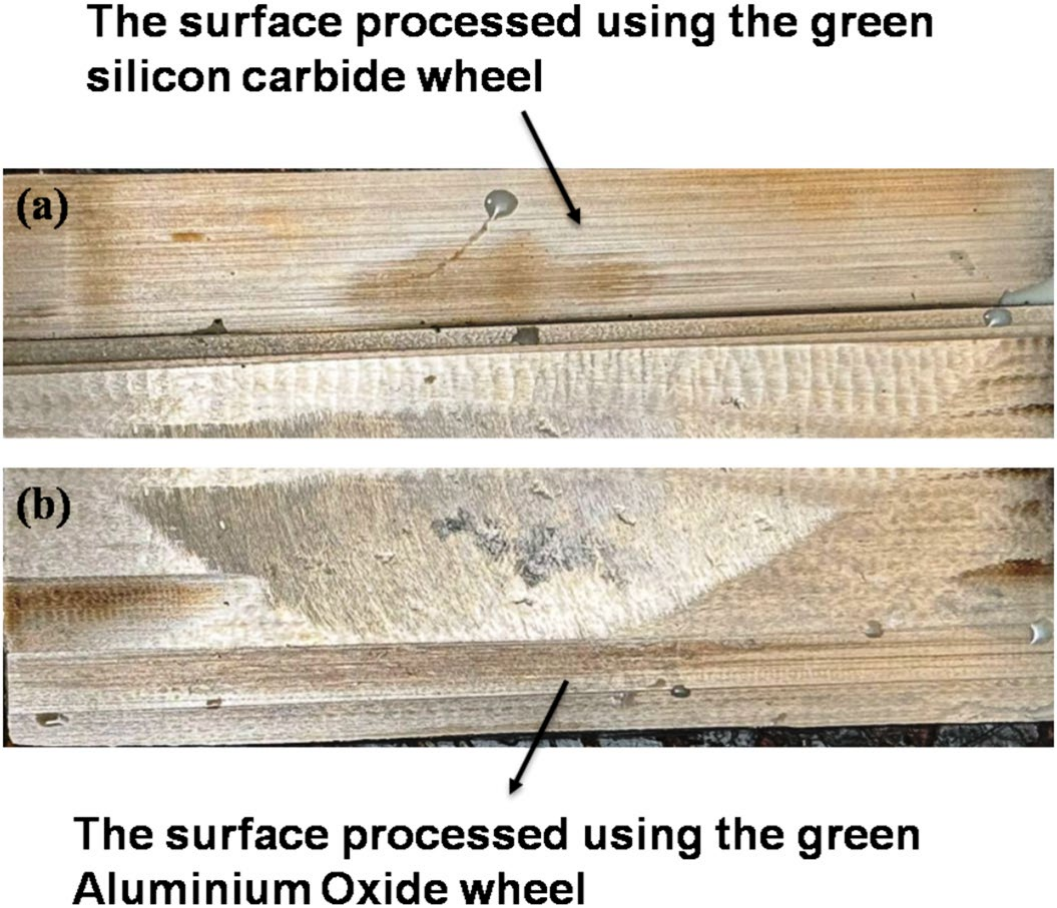
Surfaces processed using (**a**) the green silicon carbide wheel and (**b**) the aluminium oxide wheel.

The material selected for this study is Inconel 800, a nickel–iron–chromium alloy widely recognized for its high structural integrity and resistance to thermal degradation. The alloy was obtained in sheet form with dimensions of 100 mm × 50 mm × 10 mm. Its physical and chemical properties, which are suitable for high-temperature applications, are provided in Tables 1 and 2. The sheet was marked into two equal regions (25 mm each) along its 50 mm width. Two grinding wheels—aluminium oxide and green silicon carbide—were used in the grinding process. The specifications of these wheels are provided in Table 3. One region grinding process is done with a Green Silicon Carbide wheel, and the other region is done with an Aluminium oxide wheel. Figure 1 shows the macro view of the surfaces processed using (a) the green silicon carbide wheel and (b) the aluminium oxide wheel.

**Table 1:** Physical properties of Inconel 800^26^.

**Density**	**Yield strength**	**Elongation**	**Thermal conductivity**	**Ultimate tensile strength**
7.94g/cm^3	275 Mpa	45%	11.5 W/m-k	600 Mpa

**Table 2:** Chemical composition of Inconel 800^23^.

**Element**	**Ni**	**Cr**	**Fe**	**Mg**	**C**	**Cu**	**Si**	**Su**	**Al**
**Weight %**	30.86	22.721	44.007	0.430	0.081	0.474	0.260	0.006	0.201

**Table 3:** The specifications of the grinding wheels.

**Grinding wheel type**	**Abrasive material**	**Grit size**	**Hardness grade**	**Structure**	**Bond type**	**Designation**
Silicon carbide	Green Silicon carbide(Sic)	60	K	5	Vitrified	GC60 K5 VG
Aluminum oxide	Aluminum oxide (Al_2_O_3_)	60	K	6	Vitrified	RA60 K6 V206

**Fig. 2:**
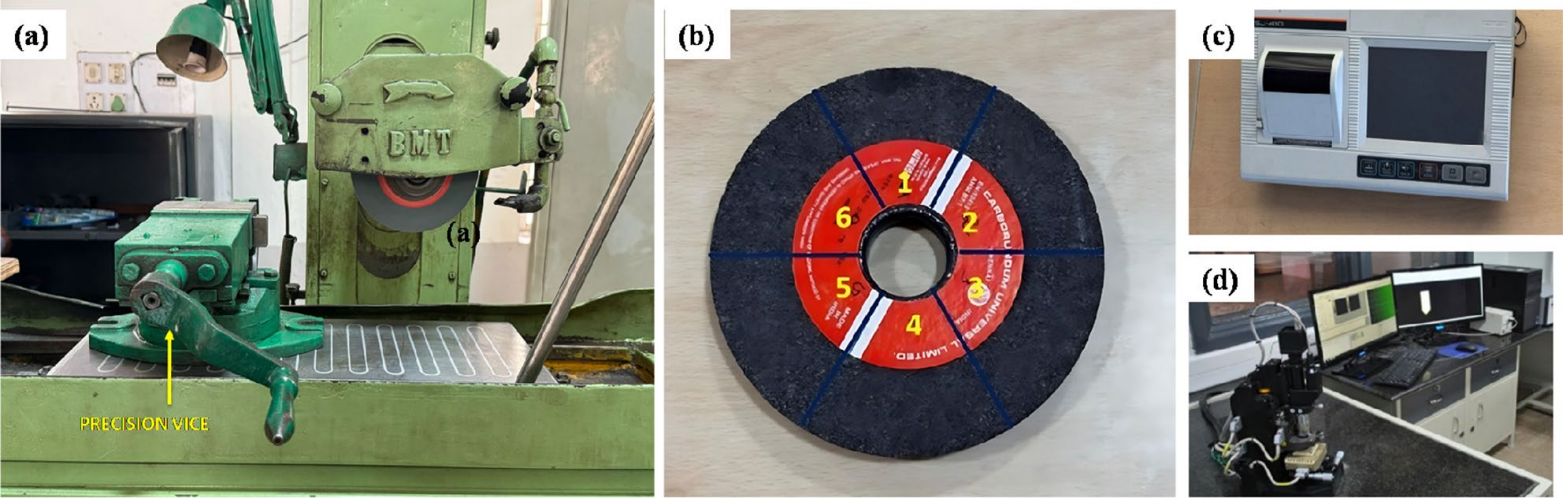
(**a**) Surface grinding machine, (**b**) Grinding wheel sectioned into six regions, (**c**) Surface profilometer, and (**d**) AFM setup.

Figure 2 shows (a) Surface grinding machine, (b) Grinding wheel sectioned into six regions, (c) Surface profilometer and (d) AFM setup. A BMT Surface Grinding Machine (Bhurji BJ-1020) was used for all grinding operations (Fig. 2a). The specifications of the grinding machine are outlined in Table 4. The Inconel 800 workpiece, being non-magnetic, was securely clamped using a precision vice instead of a magnetic chuck to ensure stable and repeatable grinding conditions. A vertical milling machine was used to make the edges of Inconel smooth and uniform for proper positioning and a tight fit into the precision vice.

**Table 4:** Specifications of surface grinding machine.

**MAKE:** BHURJI, LUDHIANA **MODEL**: BJ-1020	
Working Surface	225 x 500mm
Maximum grinding height	240mm
Spindle speed	2800 rpm
Magnetic chuck	200 x 450mm
Diamond dresser	0.5 CR
Spindle motor	0.75H.P/2800 RPM
Machine weight	700KG

**Fig. 3:**
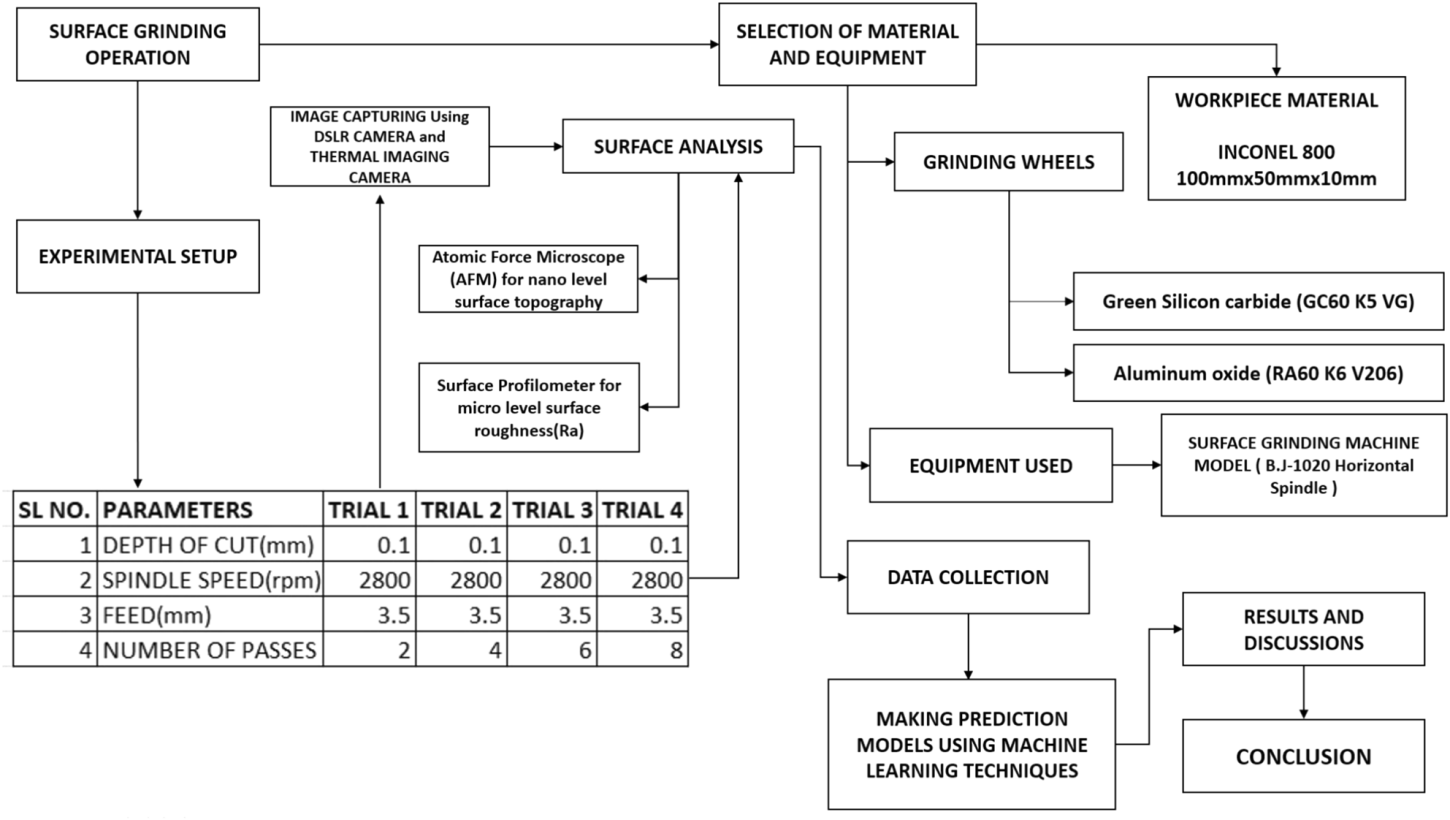
Schematic representation of the process plan used in the present work.

In this study, the grinding process involved a progressively increasing depth of cut, rising by 0.1 mm per pass, to prevent excessive heat buildup, which could potentially degrade the abrasive surface of the grinding wheel. Each wheel was pre-divided into six distinct regions to facilitate detailed image capture across various segments (Fig. 2b), enabling a more precise assessment of wear distribution. A constant spindle speed of 2800 RPM was maintained throughout the experiment. To limit thermal damage and enhance process stability, a bio-based water-oil coolant (Servo Tech Bharat) was applied consistently during grinding. Temperature variations were monitored using a thermal imaging camera (Sonel & KT-560.1) throughout the operation. After every two passes on the Inconel 800 workpiece, the process was paused to capture high-resolution images of all six wheel sections using a DSLR camera (Canon EOS 700D), which provided a clear view of the evolving wear pattern. The detailed specifications of the infrared (IR) camera are as follows: the Sonel KT-560.1 thermal camera was used, with an emissivity value of 0.30 (appropriate for a shiny metal surface), which enabled accurate thermal measurements during the grinding process. The camera’s spectral range is 7.5–14 µm, with a resolution of 384 × 288 pixels and a frame rate of 30 Hz. Surface roughness (Ra) was measured with a Mitutoyo SJ410 surface profilometer, as referenced in Fig. 2c. The entire experimental workflow, including equipment, measurement tools, material selection, and data analysis path, is systematically illustrated in the flowchart shown in Fig. 3. This cycle was repeated until a significant increase in roughness values was recorded, indicating substantial wear and increased adhesion of Inconel particles to the wheel surface. The same protocol was followed using a different grinding wheel on the remaining half of the workpiece.

Post-process evaluation was carried out using an Atomic Force Microscope (AFM), as shown in Fig. 2(d), to examine the surface roughness of the ground samples. To predict surface roughness from the acquired experimental data, multiple regression models, including linear regression, ridge regression, and random forest, were employed. The accuracy of each model was assessed using the coefficient of determination (R^2^). Furthermore, temperature data extracted from thermal images were analysed to detect heating trends across the grinding cycle. The developed model also provides estimates for wheel dressing intervals and the remaining useful life (RUL) of the grinding wheels, contributing to improved efficiency and predictive maintenance.

## Results and discussion

The grinding performance was assessed by examining changes in surface roughness across multiple passes. The recorded Ra values reflect the gradual wear of the grinding wheel and its impact on surface finish. Table 5 presents the surface roughness (Ra) measurements for each pass using the green silicon carbide and aluminium oxide wheels.

**Table 5:** Surface Roughness values Ra (µm) for Green Silicon Carbide wheel and Aluminium Oxide wheel.

**Pass number**	Green silicon carbide wheel (Ra values in µm)	Aluminium oxide wheel (Ra values in µm)
2	0.165	0.385
4	0.298	0.511
6	0.448	0.513
8	0.449	0.52
10	0.449	0.525
12	0.5	0.565
14	0.501	0.553
16	0.502	0.6
18	0.499	0.65
20	0.503	0.678
22	0.51	0.864
24	0.512	Rapid wear
26	0.52	
28	0.525	
30	0.53	
32	0.55	
34	0.61	
36	1.708	

A total of 18 Ra values were recorded for the green silicon carbide grinding wheel, as shown in Table 5 covering passes from 2 to 36. However, for the aluminium oxide wheel, only 11 readings could be taken, up to the 22^nd^ pass. This limitation arose because the aluminium oxide wheel began to experience significant heat generation and rapid wear after prolonged grinding. The excessive thermal build-up affected surface integrity, which made further passes impractical, and the wheel showed signs of degradation. Consequently, the data collection was stopped to preserve consistency and prevent further tool damage. Slight deviation from consistent increase is seen in Ra values of both the wheels since the experiment was done through manual feed. The data offer clear insights into the progressive changes in surface finish and tool performance over time. Both wheels produced consistent and comparatively low surface roughness values during the first grinding stage, indicating that the abrasive grains were cutting effectively and maintaining their sharpness without suffering severe mechanical or thermal damage. However, compared to the aluminum oxide wheel, the green silicon carbide wheel produced a notably finer finish, demonstrating its superior cutting action and initial efficacy in creating smoother surfaces.

**Fig. 4:**
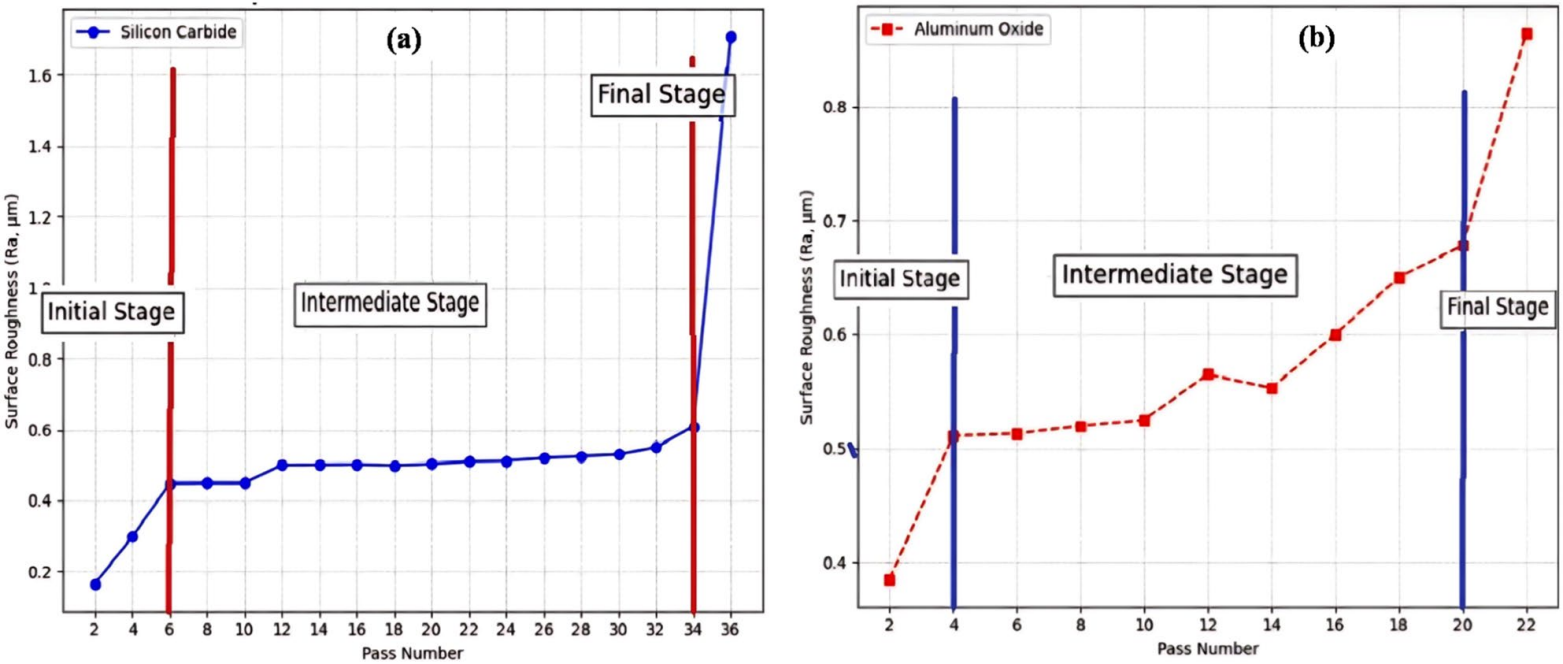
Surface roughness (Ra) versus pass number for (**a**) green silicon carbide and (**b**) aluminium oxide grinding wheels.

During the middle grinding phase, it became clear that the tools were working in different ways. The surface roughness (Ra) of the aluminum oxide wheel steadily and linearly increased, which showed that the abrasive was wearing down and the cutting efficiency was slowly dropping. Even so, the wear is predictable, which makes it fairly tolerant of moderate process changes and good for uses where consistent material removal is more important than a smooth surface finish. On the other hand, the green silicon carbide wheel kept its Ra values stable during this stage, which meant that it held onto its cutting edges better and was more resistant to wear. In line with progressive tool wear and grit dulling, the aluminum oxide wheel’s gradual increase in roughness persisted. A critical point of performance breakdown was indicated by the green silicon carbide wheel’s sharp Ra spike around the 36^th^ pass (Fig. 4a). This sudden increase is probably the result of bond failure, clogging with metallic debris, or severe abrasive grain pull-out—factors that drastically lower surface integrity and grinding efficiency. The two wheels’ wear patterns and operational suitability differ significantly when compared. Although the green silicon carbide wheel is better at providing consistently lower roughness and maintaining performance for longer, it is more likely to fail abruptly once its wear threshold is crossed. The aluminum oxide wheel (Fig. 4b), on the other hand, shows a more consistent, slow wear pattern, but because of its progressive loss of cutting efficiency, it requires more frequent dressing and maintenance.

### Temperature variations

**Fig. 5:**
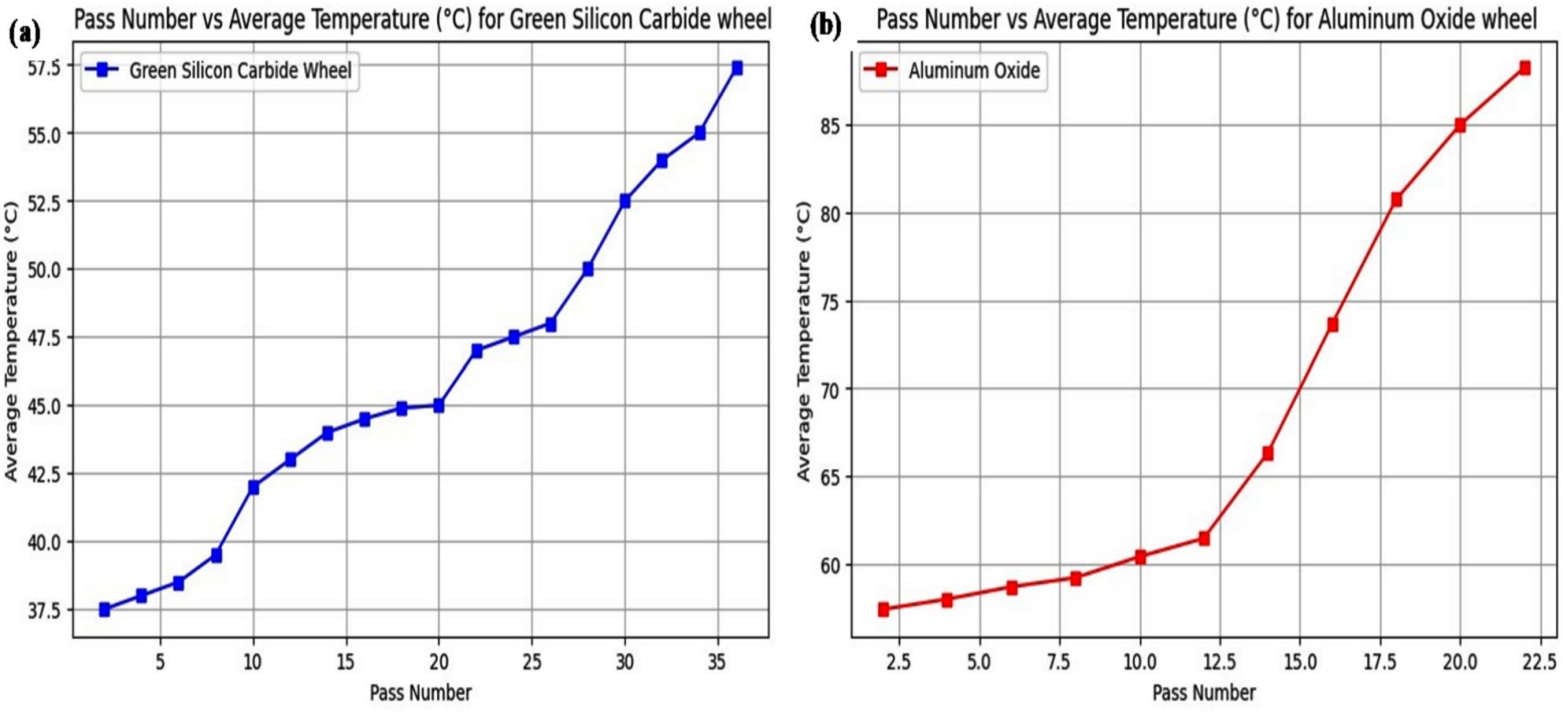
Average temperature variation with pass number during grinding of Inconel 800 using (**a**) green silicon carbide and (**b**) aluminium oxide wheels.

**Fig. 6:**
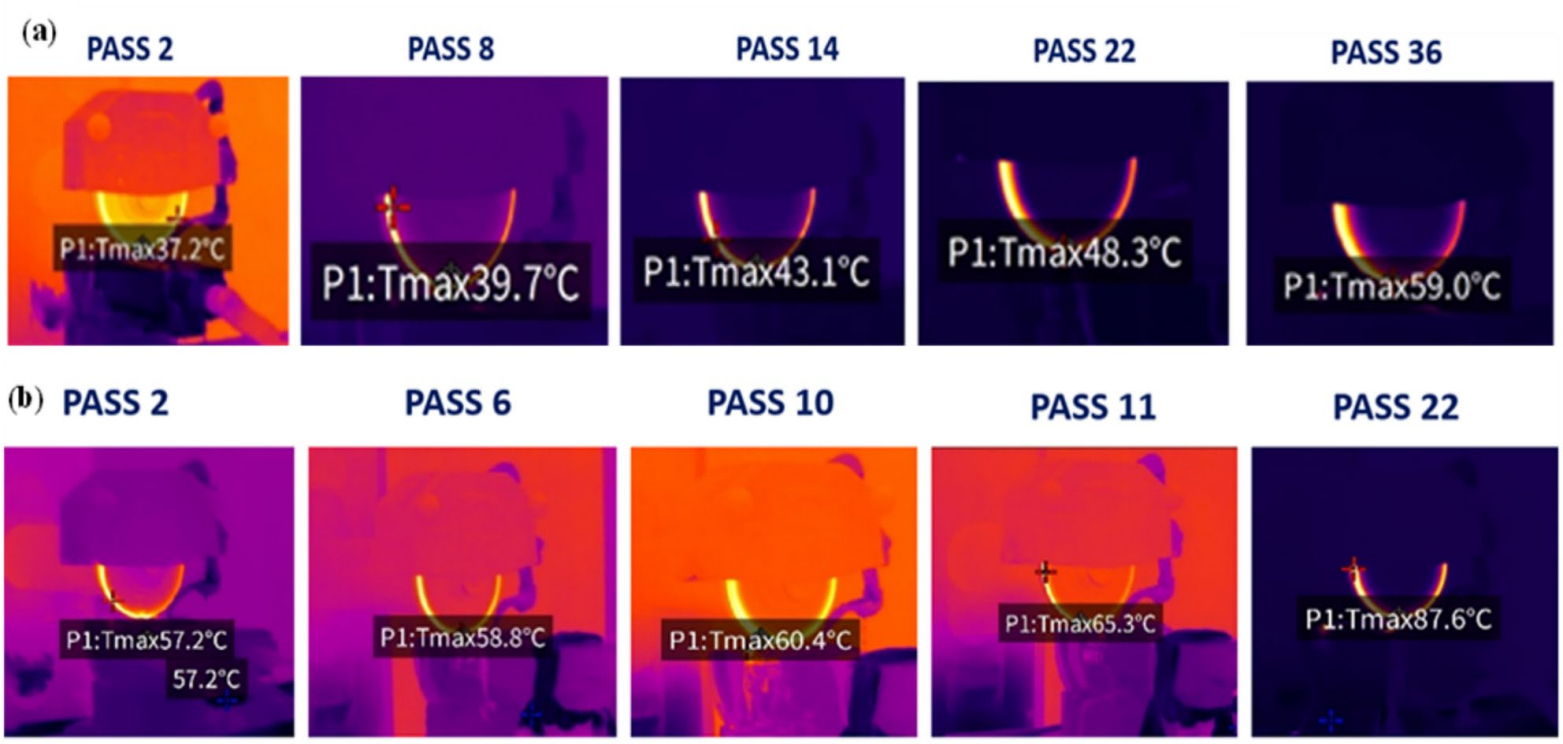
Thermal images captured during grinding of Inconel 800 using (**a**) green silicon carbide wheel and (**b**) aluminium oxide wheel at different pass numbers.

During the grinding process of Inconel 800, temperature variations were monitored to evaluate the thermal behaviour of the grinding wheels. The recorded temperature data is presented in Table 6, while the trends are visualized through plots in Fig. 5. Additionally, the variations in temperature distribution are captured through thermal images shown in Fig. 6. As abrasive grains come into contact with the workpiece, frictional forces convert mechanical energy into heat. Harder materials like Inconel 800 offer more resistance, leading to higher friction and heat generation. To mitigate thermal buildup, proper coolant flow was ensured throughout the grinding process to reduce excessive heat and protect both the workpiece and grinding wheel.

**Table 6:** Temperature variation after successive passes using green silicon carbide and aluminium oxide wheels.

**Pass number**	Green silicon carbide wheel, **Average temperature (°C)**	Aluminium oxide wheel **Average temperature (°C)**
2	37.5	57.42
4	38	58
6	38.5	58.7
8	39.5	59.22
10	42	60.42
12	43	61.46
14	44	66.38
16	44.5	73.64
18	44.9	80.74
20	45	85
22	47	88.25
24	47.5	
26	48	
28	50	
30	52.5	
32	54	
34	55	
36	57.4

Figure 5a shows that the green silicon carbide wheel maintains a steady and controlled rise in temperature, starting from 37.3 °C and gradually reaching 57.4 °C by the 36^th^ pass. This indicates efficient heat dissipation and thermal stability, which minimizes thermal load on the workpiece. In contrast, Fig. 5b demonstrates that the aluminium oxide wheel undergoes a sudden rise in temperature after the 12^th^ pass, climbing sharply to a peak of 88.25 °C**.** This rapid heat accumulation can adversely affect grinding efficiency and surface finish due to increased risk of thermal damage. The data clearly suggest that the green silicon carbide wheel is more suitable for continuous grinding operations involving heat-sensitive materials like Inconel 800, whereas the aluminium oxide wheel requires tighter control and optimized cooling to manage its higher heat generation.

The thermal images in Fig. 6a support the above findings by visually confirming the gradual temperature rise with the green silicon carbide wheel—from 37.2 °C at Pass 2 to 59.0 °C at Pass 36—along with a more uniform heat distribution. This controlled thermal behavior helps preserve surface integrity and reduce thermal-induced defects. Conversely, Fig. 6b illustrates a much more intense heat concentration during grinding with the aluminium oxide wheel, where the temperature rapidly escalates from 57.2 °C at Pass 2 to 87.6 °C at Pass 22**.** Such elevated temperatures can contribute to issues like surface burns, microcracking, and residual stress formation on the Inconel 800 surface. Therefore, the green silicon carbide wheel offers a clear advantage in thermal control**,** while the aluminium oxide wheel may only be suitable under conditions with enhanced cooling mechanisms.

### Regression model input: region-wise inconel particle accumulation

**Fig. 7:**
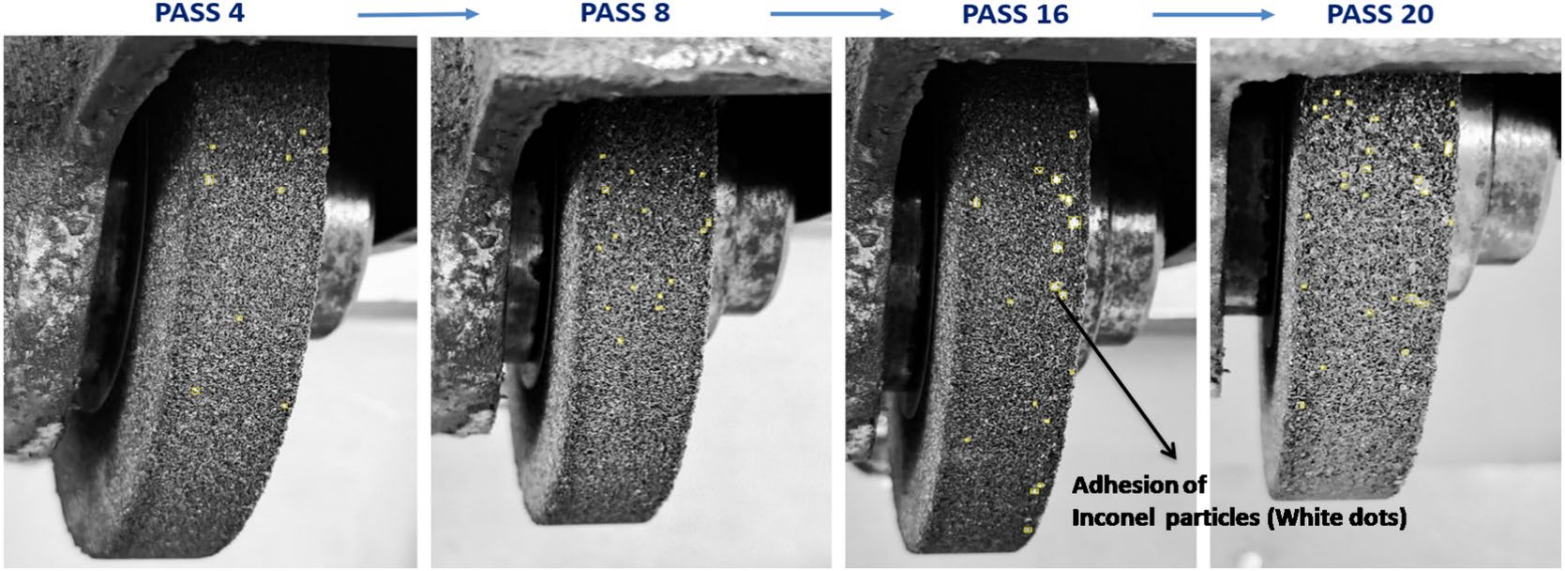
Particles embedded over successive passes in region 1 of the Aluminium Oxide wheel.

**Fig. 8:**
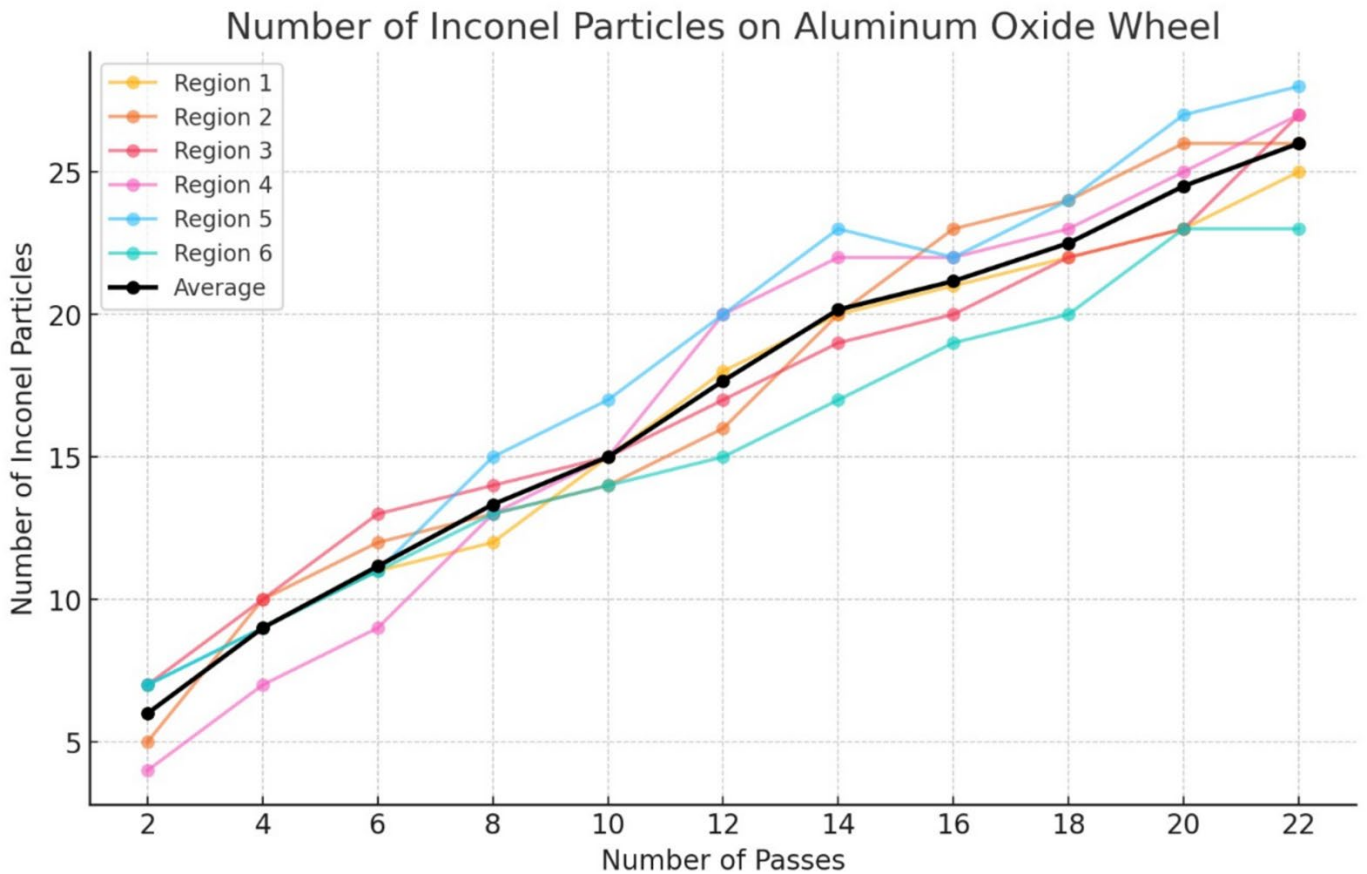
Plot of region-wise Inconel particles on Aluminium Oxide wheel across successive passes.

To develop the regression model, the aluminium oxide grinding wheel was divided into six distinct regions (Fig. 2b) and high-resolution images of each section were captured after every grinding pass using a consistent zoom level of 75%. These images were manually annotated by marking Inconel particles inside yellow boxes, which are adhered to the wheel surface as shown in Fig. 7. This systematic, region-wise annotation allowed for detailed monitoring of particle build-up over time, forming a solid foundation for reliable model development. For every pass, Inconel particles in each of the six regions were manually counted and the average of these counts was calculated to represent the overall wheel condition during that stage of the process. This averaged particle count served as the input for regression analysis, helping to model how material loading on the wheel progressed with each successive pass. The region-wise data collected is compiled in Table 7, and the trend of average Inconel deposition is shown in Fig. 8, highlighting the gradual accumulation of debris on the wheel surface.

**Table 7:** Region-wise inconel particles on aluminium oxide wheel.

**Pass**	**Region 1**	**Region 2**	**Region 3**	**Region 4**	**Region 5**	**Region 6**	**Average**
2	6	5	7	4	7	7	6
4	9	10	10	7	9	9	9
6	11	12	13	9	13	11	12
8	12	13	14	13	15	13	13
10	15	14	15	15	17	14	15
12	18	16	17	20	20	15	18
14	20	20	19	22	23	17	20
16	21	23	20	22	24	19	21
18	22	24	21	23	25	20	22
20	23	26	23	25	27	21	24
22	25	26	27	27	28	23	26

During the grinding of Inconel 800, the buildup of Inconel particles on the surface of the grinding wheel became increasingly evident as the process progressed. This adhesion was largely driven by the high thermal and mechanical loads generated at the grinding interface. With each pass, more material from the workpiece adhered to the wheel surface, reflecting a gradual and consistent material transfer. The poor thermal conductivity and strong work-hardening nature of Inconel contributed to this behaviour, resulting in elevated temperatures that promoted particle adhesion. While this trend was observed across all six regions of the grinding wheel, certain areas showed slightly higher concentrations of adhered particles, possibly due to uneven wear or localized heating effects. Around the 18^th^ to 22^nd^ pass, the distribution of particles appeared to level out, indicating a point of saturation where accumulation rates stabilized across the wheel. The average particle count per region, illustrated by the black line in Fig. 8, increased steadily and offered a dependable reference for building regression models to predict wheel wear. However, as the count approached 25 to 27 particles per region, early signs of wheel clogging became apparent. This condition negatively impacted grinding performance by reducing surface finish quality and contributing to a rise in grinding temperatures.

### Inconel particle adhesions – green silicon carbide wheel

**Fig. 9:**
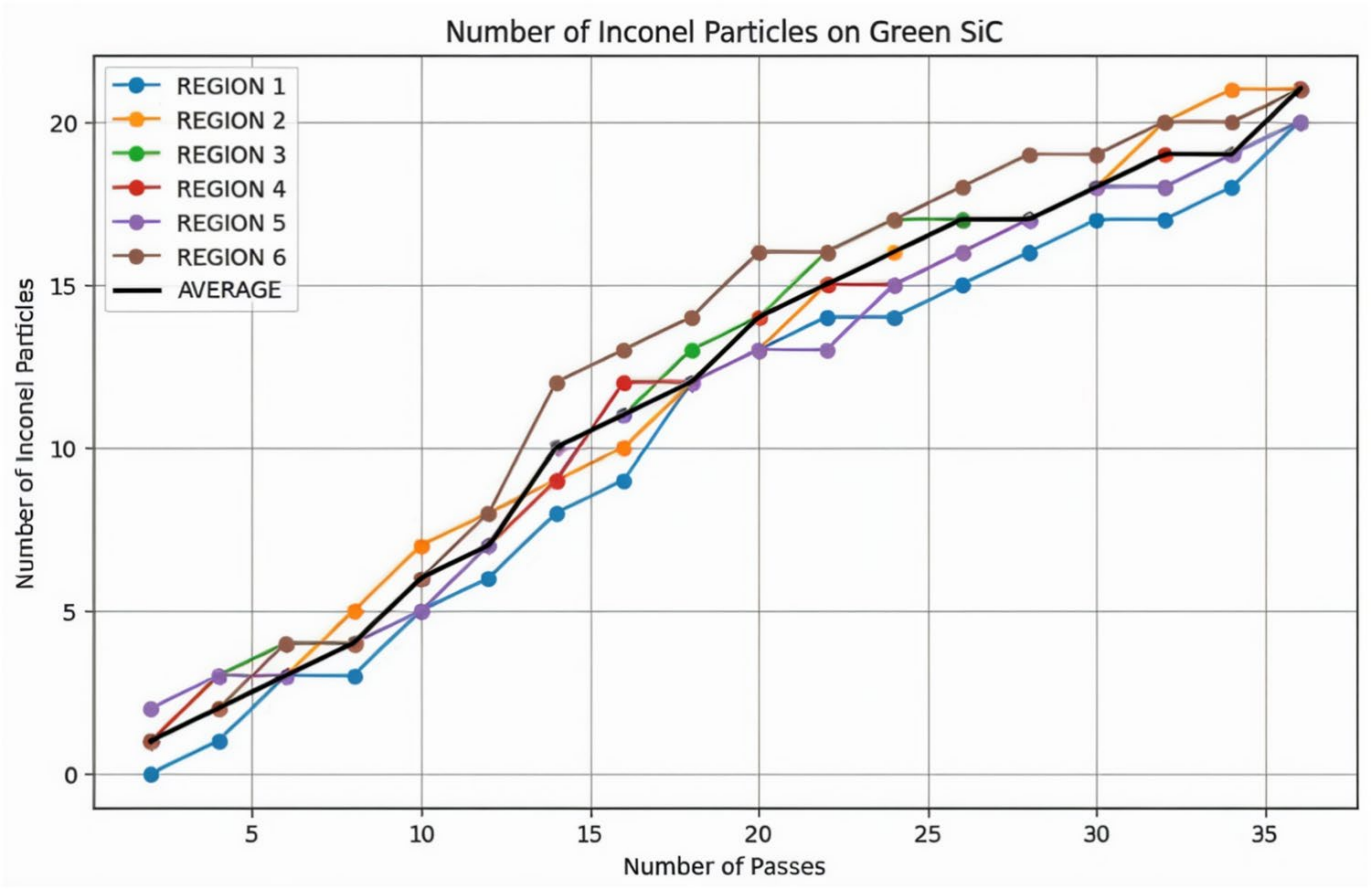
Plot of region-wise Inconel particles on the green silicon carbide wheel across successive passes.

To extend the scope of the analysis, the region-wise image annotation method initially adopted for the aluminium oxide grinding wheel was similarly applied to the Green Silicon Carbide wheel. After every two grinding passes, images were captured from six designated regions on the wheel surface, and the number of adhered Inconel 800 particles was manually counted. The resulting average particle count for each interval is tabulated in Table 8 and graphically illustrated in Fig. 9. As evident from the plot, the particle accumulation began with an average count of just 1 after the second pass and gradually increased to 21 by the 36^th^ pass. This steady and progressive rise in adhered particles suggests a more controlled and predictable wear pattern when compared to the aluminium oxide wheel, which exhibited a relatively sharper and more abrupt increase in loading during the initial stages. The consistent nature of particle build-up in the case of green silicon carbide indicates a slower rate of surface clogging, contributing to sustained grinding efficiency over prolonged operation.

**Table 8:** Region-wise Inconel particles on the green silicon carbide wheel.

**Pass**	**Region 1**	**Region 2**	**Region 3**	**Region 4**	**Region 5**	**Region 6**	**Average**
2	0	1	1	1	2	1	1
4	1	2	3	3	3	2	2
6	3	3	4	3	3	4	3
8	3	5	4	4	4	4	4
10	5	7	5	5	5	6	6
12	6	8	7	7	7	8	7
14	8	9	10	10	10	12	10
16	9	10	11	11	11	13	11
18	12	12	13	12	12	14	12
20	13	13	14	13	13	16	14
22	14	15	16	13	13	16	15
24	14	16	17	15	15	17	16
26	15	17	17	16	16	18	17
28	16	17	17	17	17	19	17
30	17	18	18	18	18	19	18
32	17	20	18	18	18	20	19
34	18	21	19	19	19	20	19
36	20	21	20	20	20	21	21

This improved performance can be attributed to the superior hardness and enhanced thermal conductivity of silicon carbide, which collectively support better abrasive retention, reduced heat accumulation and delayed grit dulling. Furthermore, the slower rate of wear allows the wheel to maintain its sharpness over a greater number of passes, minimizing the need for frequent dressing and interruptions in the machining process. These material and thermal advantages are reflected in the stable adhesion trends observed in Fig. 9, which further highlight the suitability of green silicon carbide for precision grinding tasks involving hard-to-machine materials like Inconel 800. Overall, the wheel’s performance under extended use not only ensures consistent tool condition but also contributes to improved surface finish and process reliability, reinforcing its potential for high-performance manufacturing applications.

### Regression model

In this analysis, three distinct modeling techniques were employed to investigate the relationship between pass number and surface roughness (Ra). Linear Regression was first used to establish a clear baseline, revealing a strong upward trend where each pass increased Ra by approximately 0.0226 µm. To ensure the robustness of this finding and mitigate the risk of overfitting, Ridge Regression was applied, which confirmed the stability of the linear relationship. Finally, Random Forest regression was employed to capture any complex, non-linear patterns that might be missed by simpler models. This technique proved highly effective, achieving near-perfect prediction accuracy and demonstrating its ability to model the full complexity of the process, particularly the sharp increase in Ra observed at the final pass. This multi-method approach ensured both interpretability and high accuracy in the analysis.

**Fig. 10:**
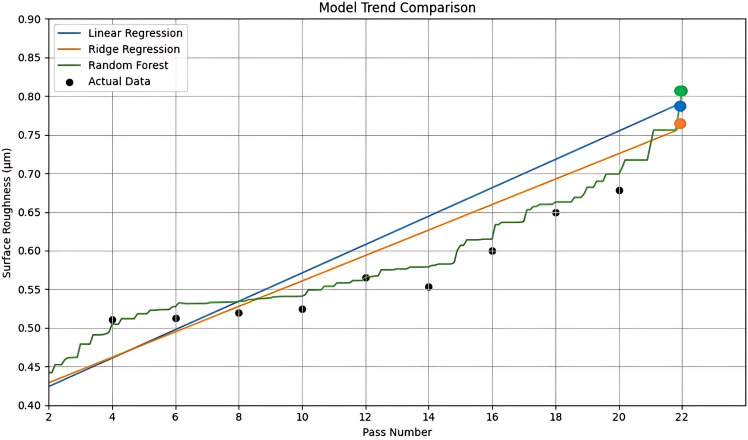
Comparison of model predictions with actual surface roughness over grinding passes of aluminium oxide wheel.

The graph shown in Fig. 10 compares the performance of three regression models: Linear Regression, Ridge Regression, and Random Forest, in predicting surface roughness as a function of grinding passes. All three models capture the general increasing trend of surface roughness along additional passes, which aligns with the experimental observations. Among them, the Random Forest model stands out by closely tracking the actual data points, especially at higher pass numbers. At 22 passes, the Random Forest predicts a roughness of 0.807 μm, closely approaching the actual value of 0.864 μm. Whereas Ridge and Linear Regression predict slightly lower values of 0.763 μm and 0.780 μm, respectively. This demonstrates Random Forest’s superior ability to model the complex non-linear relationships inherent in the grinding process. Supporting this, the R^2^ score for Random Forest was the highest at 0.9350, compared to 0.9036 for Linear Regression and 0.8839 for Ridge Regression.

As the grinding wheel begins to wear down, its ability to produce a consistent surface finish diminishes, resulting in a noticeable increase in surface roughness (Ra). To counteract this, regular dressing becomes essential as it restores the wheel’s cutting efficiency by removing embedded debris and revealing fresh abrasive grains. Understanding the Remaining Useful Life (RUL) of the wheel is important as it allows for timely maintenance, further reducing the risk of poor surface finish or tool-related defects.

According to experimental data, grinding wheel performance starts to noticeably deteriorate around the sixteenth pass. At this stage, the measured surface roughness (Ra) approaches 0.6 µm, which is frequently linked to the beginning of tool wear that degrades surface quality. After the 18^th^ pass, there is a noticeable decrease in cutting efficiency and accelerated abrasive degradation, as evidenced by a steeper increase in Ra. Ra surpasses 0.85 µm by the 22nd pass, indicating considerable tool wear and reduced material removal capacity.

These results show how important it is to regularly check the roughness of the surface to find the best time to dress the wheels. When grinding Inconel 800 with precision, it’s important to keep the surface intact. If a Ra value of 0.6 µm is used as the highest acceptable level of surface quality, the grinding wheel will work for about 16 passes. So, the wheel’s estimated remaining useful life (RUL) is about 16 passes before it needs to be dressed. After that, the quality of the surface and the reliability of the process may be affected.

## Conclusions

Under controlled conditions, grinding experiments were conducted on Inconel 800 using two medium-grit abrasive wheels—green silicon carbide and aluminium oxide. Based on the results obtained, the following conclusions were drawn:

- Measurements of surface roughness showed that green silicon carbide wheels had lower Ra values over several grinding passes, which means they were more resistant to wear. On the other hand, the roughness of the aluminum oxide wheels kept going up, which meant that the tools were wearing out faster.
- For the aluminium oxide wheel, region-wise manual annotation was carried out to quantify the number of Inconel particles deposited on the wheel surface. A progressive increase in particle adhesion was observed with each pass, highlighting potential clogging and a decline in grinding efficiency.
- Regression models were developed to predict surface roughness using inputs such as average temperature, depth of cut, and particle accumulation. Among the models tested, Random Forest regression showed the highest accuracy with an R^2^ score of 0.9350.
- AFM analysis provided valuable nanoscale insights, showing that while Aluminium Oxide exhibited smoother macro surfaces (Ra = 0.864 µm), Green Silicon Carbide achieved a superior nano finish (Ra = 0.991 nm), emphasizing the importance of multi-scale surface characterization.
- Thermal analysis showed higher heat buildup in aluminium oxide grinding, reaching up to 88.25°C, while green silicon carbide maintained lower temperature levels, indicating better thermal stability and reduced risk of thermal damage to the workpiece.
- Based on regression trends and thermal behavior, the recommended dressing interval for aluminium oxide was identified around 16–18 passes, beyond which surface roughness and heat generation significantly increase, reducing grinding efficiency.

## Data Availability

The data presented in this study is presented in the manuscript.
